# Development of a decision‐support framework to support professionals and promote comfort among older hospital inpatients living with dementia

**DOI:** 10.1111/hex.13922

**Published:** 2023-11-27

**Authors:** Nathan Davies, Emily West, Ellen M. Smith, Victoria Vickerstaff, Jill Manthorpe, Malvi Shah, Greta Rait, Jane Wilcock, Jane Ward, Elizabeth L. Sampson

**Affiliations:** ^1^ Department of Primary Care and Population Health, Centre for Ageing Population Studies, Royal Free Campus University College London London UK; ^2^ Marie Curie Palliative Care Research Department University College London London UK; ^3^ South West London and St George's Mental Health NHS Trust London UK; ^4^ NIHR Applied Research Collaborative (ARC) South London King's College London, Strand London UK; ^5^ NIHR Policy Research Unit in Health and Social Care Workforce King's College London, Strand London UK; ^6^ Patient and Public Involvement Representative Lonodn UK; ^7^ Department of Psychological Medicine, Royal London Hospital East London NHS Foundation Trust London UK

**Keywords:** decision making, dementia, hospitals, patient comfort

## Abstract

**Background:**

Acute hospital wards can be difficult places for many people living with dementia. Promoting comfort and wellbeing can be challenging in this environment. There is little evidence‐based support for professionals working on acute care wards on how to respond to distress and maximise comfort and wellbeing among patients living with dementia.

**Objectives:**

Our overall aim was to codesign an evidence‐based easy‐to‐use heuristic decision‐support framework, which was acceptable and practical but acknowledges the complex and acute nature of caring for patients with dementia in the hospital. This paper presents the development process and resulting framework.

**Methods:**

A codesign study was informed by data from (1) a literature review of the care and management of people living with dementia in acute hospitals; (2) a cohort study of comfort and discomfort in people with dementia in acute hospitals; and (3) interviews with family carers and health care professionals. We synthesised evidence from these data sources and presented to key stakeholders through codesign meetings and workshops to produce our decision‐support framework.

**Results:**

The framework consists of a series of flowcharts and operates using a three‐stage process of: (1) assess comfort/discomfort; (2) consider causes of discomfort; and (3) address patient needs to manage the discomfort.

**Conclusion:**

Working with key stakeholders, synthesising diverse quantitative and qualitative evidence to build a clinical framework is a feasible approach to help address the needs of patients living with dementia in an acute hospital setting. The result is a framework which is now ready for evaluation and implementation.

**Patient and Public Contribution:**

We worked closely with people living with dementia and family carers throughout this study, including the development of the study protocol with input on study development and design, through to inclusion in stakeholder workshops and codesign of the decision support framework.

## 1 INTRODUCTION

Dementia affects nearly half (42%) of acute hospital inpatients in the United Kingdom, of these nearly half have moderate or severe levels of cognitive and functional impairment.^1^, ^2^ The number of people living with dementia worldwide is expected to increase as more people survive into old age.^3^ Most people living with dementia have other long‐term health conditions or comorbidities, and have high rates of health care usage including acute inpatient stays.^4^ Acute hospital wards can be difficult settings, particularly for those patients living with dementia, and hospital admissions often exacerbate symptoms and behavioural distress associated with dementia.^5^ These patients need higher levels of nursing care, have longer stays, experience greater functional decline and are more at risk of delayed discharge than the general patient population.^6^ These experiences affect not only patients and family carers but practitioners who need to support patients in distress^7^ and the wider system through increased costs and health care use. Many concerns have been raised regarding low levels of knowledge and skills in dementia care amongst acute hospital staff in the United Kingdom.^8^ Family carers show high levels of dissatisfaction at a lack of understanding of the needs and vulnerabilities of patients with dementia,^9^ and inadequate person‐centred approach and a lack of compassion, particularly with regard to personal care and communication with the patient with dementia which is often functional and task orientated.^8^, ^10^

There is a lack of evidence‐based best practice principles and guidelines in acute hospitals for dementia care, which reinforces staff uncertainty when caring for patients with dementia.^10^ Guidelines on dementia care are inconsistently developed and available in individual hospitals, however, there is a lack of practical support for the multidisciplinary team in caring for patients with dementia, including assessing and addressing complex and multiple needs in a busy and sometimes under‐resourced environment. Assessments often consist of standardised measures which help identify problems (i.e., Abbey Pain Scale [APS]), however, do not provide advice on how to manage identified needs. Acute hospital staff have raised particular concerns about assessing and managing agitation and delirium in patients with dementia who were agitated.^11^, ^12^

The UK Prime Minister's 2020 Dementia Challenge called for ‘every person diagnosed with dementia having meaningful care’.^13^ Little research has considered how best to improve care for patients living with dementia in acute hospitals,^14^ but a systematic review identified factors that may increase comfort for patients in this setting, including minimising external environmental stressors such as overstimulation through noise and light and attending to internal stressors such as pain and hunger.^11^ Providing an easy‐to‐use decision support framework to help guide staff in delivering person‐centred and holistic care has the potential to improve hospital care and promote nonpharmacological methods for managing distressing symptoms and distress associated with a hospital stay. Other studies have developed easy‐to‐use heuristics/rules of thumb to support professionals' care decisions for people living with dementia.^15^, ^16^ Heuristics/rules of thumb are broad principles, which prompt users to think about the problem, solutions, and lead to action.^17^ They reflect the innate thought processes of experts, making this implicit knowledge experts have developed over many years of experience, explicit for all to know. They are represented as schematic patterns or flowcharts which prompt users through a series of thought processes, options, and decisions. These have been successfully implemented in health and social care settings including care home training programmes,^18^ however these heuristics/rules of thumb have not focused on acute care or on discomfort and distress.^15^, ^16^ These previous rules of thumb are broader and designed to be used across different settings. Rules of thumb in dementia care have shown high acceptability among health care professionals, and provide a structure for decision making, reducing complexity and offering reassurance and instilling confidence in professionals.^15^ However, previously developed rules of thumb lack specific consideration in the context of busy acute medical and surgical wards where time is limited and staff may not have any previous knowledge of the patient.

The overall aim of our study was to codesign an evidence‐based rules of thumb decision‐support framework which is easy‐to‐use, acceptable, and practical, whilst acknowledging the context including the complexity and acute nature of caring for people living with dementia in hospital. The framework would support acute hospital staff and family carers in identifying discomfort and distress, maximising patient comfort (and thus wellbeing) and reducing behaviours that challenge others or are distressing to people living with dementia in this setting. This paper presents and discusses the development process and the decision‐support framework.

## 2 METHODS

### 2.1 Design

A codesign study underpinned by O'Cathain et al.'s^19^ taxonomy of approaches to intervention development, following similar studies in dementia care.^20^, ^21^ We followed the synthesis actions described by O'Cathain et al.,^19^ these included: understanding the problems which need to be addressed, assess the causes of the problems, understand the wider context (here of the acute hospital), identify possible ways of making changes to address the problems, and identify evidence of effectiveness of interventions for the identified problem (here comfort and discomfort in acute hospitals).^19^ To operationalise these actions and inform the codesign of the framework, we used three sources of information from earlier phases of this study—(1) a cohort study of comfort and discomfort in people with dementia in acute hospitals to understand the context and problems to be addressed; (2) interviews with family carers and health care professionals to understand in more depth the challenges and context and possible changes; and (3) a literature review of the care and management of people living with dementia in acute hospitals to identify evidence to address the problems. This information was supplemented with relevant reviews of the literature and guidance from leading organisations. The data sources were synthesised and presented to key stakeholders through codesign meetings and workshops.

### 2.2 Data sources and earlier phases of the project

#### 2.2.1 Cohort study

We conducted a prospective cohort study of 64 patients (aged over 65 years) living with dementia on acute hospital wards in two London hospitals. Participants were mainly female (58%), of White ethnicity (57%), and a large proportion in the older age category (54%, aged 75–84 years). We collected data on pain, delirium, comfort, psychiatric symptoms, and the care environment using the following validated measures:

1. Memorial Delirium Assessment Scale.^22^
2. Sources of Discomfort Scale.^23^
3. Pain Assessment in Advanced Dementia (PAINAD).^24^
4. APS.^25^
5. Symptom Management‐End Of Life in Dementia.^26^
6. Neuropsychiatric Inventory (NPI).^27^
7. Cohen‐Mansfield Agitation Inventory (CMAI).^28^

This cohort study ran in parallel to the codesign and therefore we conducted regular interim descriptive analysis to inform the codesign of the decision support framework. From the interim analysis we identified the main challenges, namely pain (40% at rest, 47% during movement) disorientation (mean: 2.4 indicating moderate disorientation), constipation (44%), sleepiness (57%), lack of movement (48%), anxiety (>50%), agitation (60%) and depression (<60%) (see Table 1). Hospital wards were at times noisy (mean: 23.78 dB/1–48 dB) and warm (mean: 24.65°C/19.2°C–38.9°C) which may create further difficulties. Staff‐reported scales found many patients were restless, and seemed distressed as measured through several of the scales including PAINAD, CMAI and NPI. Staff found this hard to manage, especially if it led to violence or verbal aggression. A final descriptive data analysis of the full data set was conducted in April 2023 to refine the framework. The final analysis will be published separately. The results from the cohort helped us to understand the key symptoms and challenges to be addressed in the decision support framework and what to present to stakeholders in workshops.

**Table 1:** Summary matrix of evidence to inform heuristic development.

Decision toolkit section	Systematic review^14^ evidence and additional resources	Interview data	Cohort data and scales	Codesign recommendations
Communication	Our systematic review of interventions to improve care and management for people with dementia in hospitals identified music therapy, multimodal‐comprehensive care, person‐centred care, and family‐centred function‐focused care interventions improve staff knowledge, competence, efficacy, and communication.^14^ Several organisations in the United Kingdom including Alzheimer's Society and Social Care Institute for Excellence have provided top tips on communication with a person living with dementia including: short, simple sentences; communicating clearly and calmly. Try to communicate with the person in a conversational way, rather than asking question(s).^29^, ^30^ Following guidance from Mental Capacity Act (2005)^31^ and National Institute for Health and Care Excellence (NICE) guideline [NG97] Dementia: assessment, management and support for people living with dementia and their carers (2018)^32^; communication is vital, and everything should be done to ensure effective communication, adapting to the needs of the individual.	Communication was felt to be vital in ensuring comfort in patients with dementia. Communication is often complex and staff were reliant on nonverbal signs of discomfort/comfort such as facial expression, behaviour and gestures. Staff used simple language when talking to their patients and advised slowing down, using pictorial information and trying to ask pointed questions about sources of discomfort. Sometimes, the manifestation of the distress would be pointing or gesturing towards something causing the distress or was a direct response to an immediately identifiable trigger. Staff members frequently highlighted the need to know their patients, their backgrounds and their families to ensure their needs were met. They advised getting as much information about the patient from their family and friends to understand them and their preferences and to use this information to guide their care of the individual. Carers reported to minimise distress, it was important not to continually ask the person living with dementia for their agreement or to try to reason with them, as this could lead to an argument and more distress. Important to use firm and reassuring language.	Features of APS and PAINAD highlight areas to consider for communication including vocalisations, facial expressions, body language, behavioural changes. On the PAINAD the pain facet with the highest rate of ‘severe’ observation was facial expression, this was mirrored in the APS.	Communication is important to consider throughout not only second languages, but also aids to support communication for example hearing aids, but also dentures. This should be provided as a clear message to users of the framework from the start. Communication should consider not only verbalisation but think about faces and body language.
Assessment	Clear communication, aligning with person‐centred dementia care is vital, including the person with dementia where possible but also the family carer.^15^	Interviews highlighted the need to understand comfort is more than the absence of pain, comfort is about ensuring that a person's needs are met from a physical and a psychosocial perspective. A holistic approach was felt to be required to identify sources of discomfort in a patient. When challenging behaviour was experienced, staff advised methodically excluding causes of discomfort, checking through each potential cause of discomfort and addressing them. It was important to provide verbal reassurance to the patient. Using simple speech and reviewing patterns of behaviour (such as ABC charts) were felt to be helpful and again, knowing a patient's interests and preferences is vital (e.g., ‘This is me’ document). Staff reported being able to identify discomfort in patients due to a deviation from baseline behaviour, including patients ‘shouting, crying, sometimes actually physical aggression towards others’ or becoming more tearful and withdrawn. Participants could tell if a patient was comfortable through observing their body language. Comfortable patients would have relaxed facial features, would smile and appear happy.	Several scaled/outcome measures used in the cohort study provided ideas of what to consider during assessments—including APS, PAINAD, SODS, NPI, MDAS, SMEOLD and CMAI items. Analysis demonstrated a variety of unmet needs and high symptom burden across communication (31% patients swore or cursed at staff, 26% repeated requests for attention), behaviour (16% kicking, 18% reported wandering or pacing), and emotional distress (60% had periods of agitation, and over 60% feeling depressed)—suggesting key areas for assessment.	Assessment follows throughout all sections of the heuristics. Key points to consider include—communication is key, how is the individual communicating and behaving? Ensure you observe the patient. What is the patient's baseline or normal for them? Ensure you talk with the patient. Talk to the family to understand more about the individual and learn from them about what is their routine or normal for them. This may include what do they have for breakfast, or what do they normally look like (i.e., do they shave daily, do they wear makeup?). Stakeholders highlighted that framework should consider not only what happens when people are not comfortable, but also consider what is happening when they are happy and feeling comfortable. This will support learning for those providing care. In particular family carers could support with this by adding notes to the rules of thumb.
Causes—pain	Pain is often under detected in people with dementia, however it can be very common and difficult to manage.^33^ There are a variety of tools and measures to support pain assessments^34^ including PAINAD and APS with elements were incorporated into the pain rule of thumb—that is, facial expressions, body language.	Participants were keen to stress although pain is a common cause it is not the only cause. Professionals however did flag how dental pain is often forgotten but a common cause. Carers highlighted pain may not be easy to always spot, but need to consider causes including osteoarthritis and arthritis.	Pain was not the most common cause of discomfort, and most patients across pain scales scored low, with 40% indicating pain at rest and 47% during movement on the PAINAD scale. The SMEOLD measure found 73% reported being in pain for at least some of the stay in hospital. Items of PAINAD; APS; PainChek provide examples of indicators of pain incorporated into the rules of thumb.	Speaking with family or advocate should be central, part of the process rather than a side note. This can be a process point—talk to family, ask if there are other underlying conditions that may cause pain. Highlighting dental problems and oral hygiene was key and was welcomed.
Causes—physical needs	Supporting patient mobility is important as it enables patients.^35^ Eating and drinking difficulties, pressure sores/ulcers, urinary tract infections, and multimorbidity are all common challenges in older people with dementia admitted to acute hospital wards.^36^, ^37^, ^38^	Participants described how comfort is ensuring that a person's needs are met. They also described how discomfort can mean pain but can also encompass other physical needs including hunger/thirst, pressure areas, poor sleep, feeling too hot or cold.	Physical discomfort was the main cause of discomfort on the SODS with most common being sleepiness (57%) and constipation (44%). Items from SMEOLD and SODS were incorporated.	Mobility is important—are they able to stand and move about this will help with assessment. Ward staff should not be too concerned with patients moving around, this will be good for the patient and allow them to socialise as well as become tired and help with disrupted sleep. In addition to considering physical needs which cause discomfort need to ensure consider positive side of moving—prevention of pressure sores/ulcers.
Causes—emotional wellbeing	Resistance has been described on hospital wards—including resistance to care, medications and food. This is often misinterpreted as the person lacking capacity and a feature of a dementia diagnosis.^39^ Interactions are important between staff and patients, considering language used. A lack of decision making capacity should not be assumed as per the Mental Capacity Act 2005.^31^ Anxiety, depression and apathy are common among older people with dementia in hospital.^38^ Various approaches have been developed to promote person‐centred care and getting to know the patient including: 10‐Things; This is Me; and My Care Matters.	Carers discussed a wide breadth of emotions leading to distress including: anxiety, anger, embarrassment, disgust, frustration, and fear.	The most common symptoms were agitation (60%), anxiety (53%) and depression (63%) and occurred frequently. We incorporated items from NPI; CMAI; and SMEOLD.	Following on from the discussions of communication section—it was felt interactions were a core feature of emotional wellbeing. Anxiety was discussed as being heightened in hospitals—both from patient and carer perspectives—this needed to be highlighted and addressed. Social needs were important considering what was available on hospital wards—including books and magazines. Knowing about the patient was key—stakeholders highlighted the need to use existing resources such as the ‘10‐things’ or ‘This is me’ documents.
Causes—Environment	Hospital environments can be chaotic, loud and disruptive for patients with dementia. It is important to enable a place of safety and opportunity for social interactions.^40^ Opportunity for quiet space and areas are important in minimising disruption from other patients. Homeliness is considered important by people with dementia and their family carers, this includes light, familiar furniture and an sunny outlook.^5^ Decline of the patients condition in hospital may be in part caused by the environment.^41^	Participants discussed how the environment can affect the comfort of a person with dementia. A hospital ward is not home and, although adaptions are made, they are not usually ‘homely’ and are often unfamiliar. Other patients may cause distress and the environment can be busy and confusing. This is an area that staff are mindful of but felt unable to address due to the nature of the hospital environment.	Items form TESS‐NH were considered; and noise, temperature and light levels using a Multifunctional Environment Measuring Monitor—an industry certified metre and observational reports from the research team. Average temperature was 24.65°C, as recommended by the USA CDC,^42^ however the range demonstrates extreme values in some instances (19.2°C–38.9°C). Sound was recorded with a mean of 23.78 dB, however ranged from 1 to 48 dB, while the WHO recommend noise should not exceed 30 dB.^43^ Lighting level averaged 8.77 lux however ranged from −0.4 to 118.7 lux. However general lighting is recommended at 100 lux with 300 lux for general nursing care, and 5 lux for night light.^44^ There was frequent use of restraints, with little to no home like features.	Overall stakeholders agreed that people are often moved around hospital wards, and this causes disorientation and should be highlighted in the heuristic. Using personal belongings will help individuals be able to recognise their bed area. ‘Home like’ is important, and this includes having a window they can see out of to be close to nature. Environmental measures from the cohort study should be included/considered—that is, temperature, sound, lighting.
Causes—Delirium	Dementia is the strongest risk factor delirium, if a patient is appearing confused THIINK delirium The NICE recommend a tailored multicomponent intervention. The Hospital Elder Life Programme is effective in reducing incidence delirium, the programme includes daily visits, orientation, therapeutic activities, sleep enhancement, early mobilisation, vision and hearing adaptation, fluid repletion, and feeding assistance. Additions have included infection and management of constipation, pain, and hypoxia.^45^	Delirium was not specifically discussed in interviews beyond being something to consider in assessment of causes.	Items from MDAS were incorporated. Challenges highlighted in the cohort included sleep–wake disturbance (mean: 1.5 indicating mild‐moderate disturbance) and disorientation (mean: 2.4 indicating moderate disorientation).	It was not surprising this had not come up in interviews or the review as it is often neglected or poorly considered. Stakeholders highlighted the need to link to existing resources which had been developed—including PINCHME.^46^ A formal assessment is needed if delirium is suspected—consider MDAS, bCAM, MMSE, 4AT. It was important to consider an individual's baseline—for example shouting may be normal for some individuals, or these may be their usual sleeping habits. Important to remember to speak to family and communicate with the patient.

Abbreviations: 4AT, Rapid Clinical Test for Delirium^47^; APS, Abbey Pain Scale^25^; bCAM, Brief Confusion Assessment Method^48^; CMAI, Cohen Mansfield Agitation Inventory^28^; MDAS, Memorial Delirium Assessment Scale^22^; MMSE, Mini Mental State Examination^49^; NPI, Neuropsychiatric Inventory Questionnaire^27^; PAINAD, Pain Assessment in Advanced Dementia Scale^24^; SMEOLD, Symptom Management‐End Of Life in Dementia^26^; SODS, Sources of Discomfort Scale^23^; TESS‐NH, Therapeutic Environment Screening Scale for Nursing Homes & Residential Care.^50^

#### 2.2.2 Interviews with family carers and health care professionals

Finally, we conducted 12 semi‐structured interviews with current (*n* = 8) and former family carers (*n* = 4), ranging from 48 to 73 years of age, eight females, and including a spouse (*n* = 1), adult children (*n* = 9), sibling (*n* = 1) and grandchild (*n* = 1). Six interviews were conducted with staff with experience of working with patients living with dementia in acute hospitals including, four females, with an age range 33–58 years old. Professionals were occupational therapists (*n* = 4), a mental health nurse (*n* = 1) and a psychiatrist (*n* = 1). These interviews helped us to identify current practice and gain an in‐depth understanding of challenges and approaches to identifying discomfort and strategies used to promote comfort. Family carers were recruited through Twitter, hospitals taking part in the cohort study, and carer organisations. Professionals were recruited via hospitals taking part in the cohort study, and snowballing methods. All recruitment was supplemented with snowballing methods. Interviews were transcribed verbatim and analysed using codebook thematic analysis.^51^

Interviews found that comfort is more than the absence of pain, comfort is about ensuring that a person's needs are met from a physical and a psychosocial perspective.

Could be physical, could be emotional, could be it is more than pain, could be emotional, umm Yeah. Oh, I see. Yes. There you go. More than pain. (Psychiatrist)

A personal approach to care is crucial to providing comfort to people living with dementia. Importantly, family carers argued that the person living with dementia needs to feel in control when receiving care. Both groups of participants highlighted the need to know patients, their backgrounds to build a strong relationship with them and ensure needs were met.

[the care worker] had this bell and he brought a bell to her and he said, look, you hold on hold on to this bell while I'm changing you and everything. And if you feel discomfort ‐ it's best if you ring the bell. Then I know to stop or I need to finish or whatever. So, I thought that was a really good move. That she felt that she had some control over what was being done to her. (Family carer M5)

So I think for me it would be, you know, understanding the patient. So for example, if somebody is very confused and unable to express their needs, I might want to get to know this patient a bit more, which can involve, you know, involving family members, getting collateral information and trying to understand background information, life history and so gathering information from other people who have previously known the patient to understand what's actually, happening or his likes or dislikes. (Mental health nurse)

Psychological distress was a common challenge that participants discussed manifesting as restlessness, agitation and becoming withdrawn:

we don't want her to go to the hospital because she doesn't And handle that well, at all. And I found it quite distressing as well because she would be lying in bed, upset, talking to herself, agitated, her hands … and she was going through a stage when she would smack her head because … she couldn't think. (Family carer E3)

Communication was described as complex and a key challenge staff faced, they described how they relied on nonverbal signs of discomfort/comfort such as facial expression, behaviour and gestures.

I mean, I actually think it's easier than you think with this patient group. You are using a lot of, what I would call, intuitive care. You're trying to predict to begin with. You're looking at those kind of non‐verbal signs you know? Agitation, pacing, fiddling. Things like that and also you can see in facial expression, gesture. (Occupational therapist 1)

Staff often talked about mitigating the lack of communication abilities of patients, through talking to their families to help them problem solve challenges and understand how the patients were feeling or what they were experiencing. The below extract highlights how staff were often faced with behaviours that challenge, including aggression:

we tried a lot of things but he was quite physically aggressive to staff and it was quite unpredictable. He was extremely nonverbal. He couldn't understand. He had expressive and receptive kind of language difficulties, so everything had to be anticipated for him and it was speaking to his wife. And I mean we went down the traditional route of kind of looking at antipsychotic medications and depressants, so it was quite medication heavy because the risks were high. But he had no cartilage in his left knee and so he'd meant to have a knee operation. But then his dementia progressed too quickly to for him to kind of tolerate the surgery. So, and that was something when we looked at his pain relief. It was. he wasn't taking any, and he, according to his wife, he'd always had a really high pain threshold, so he wouldn't be able to necessarily indicate when he was in pain. (Occupational therapist 2)

Staff and families provided an overview of common causes of discomfort including constipation, pain, psychological symptoms such as depression or hallucinations, pressure ulcers, poor sleep, feeling too hot or cold, feeling bored, problems communicating needs and difficulties with the environment. Dental pain was a particular concern because diagnosing and treating it is difficult.

A hospital ward is likely to be much busier. And there's going to be more noise. There's going to be more observations being taken up. There's going to potentially be people that are very, very unwell, and so there's going to be kind of other people that are quite distressed. (Occupation therapist 2)

I mean, one of the things which in my past experience has been a big uh, thing to be very mindful of with pain is toothache. You know people with dementia trying to do dental hygiene‐ it's really hard and actually things like that are very difficult to spot. And actually we know what it's like with toothache‐ so painful. And actually, when you can't communicate that … So again, I think it's really being mindful of doing almost like that unmet needs kind of checklist in your head. (Occupation therapist 1)

Staff felt their understanding of discomfort was learned through experience although it could be innate or intuitive for some, highlighting the potential for a ‘rule of thumb’ for those less experienced in working with patients living with dementia.

#### 2.2.3 Systematic review

Through a systematic review we aimed to identify and assess the effectiveness of interventions designed to improve the care and management of patients living with dementia in acute hospitals,^14^ We found evidence that multisensory behaviour therapy reduces what are sometimes termed behavioural and psychological symptoms of dementia, multidisciplinary programmes reduce postoperative complications^52^ and that robot‐assisted therapy,^53^ music therapy,^54^ multimodal‐comprehensive care, person‐centred care^55^, ^56^ and family‐centred function‐focused care interventions^57^ improved patient outcomes, staff knowledge, competence, efficacy and communication. However, there was only low to very low‐quality evidence thus limiting the ability to make clinical recommendations.^14^ Hence, novel approaches such as the use rules‐of‐thumb might help guide care and decision making.^17^

#### 2.2.4 Synthesis of evidence

The qualitative interviews and cohort study enabled us to explore in more depth the problems facing people living with dementia in acute hospitals. We considered the design of the intervention guided by some of the design actions identified by O'Cathain et al.^19^ to inform our stakeholder workshops. This included possible ways of making changes to address the problems, considering the real world delivery of any intervention we developed, beginning to design and create our intervention through generating ideas about solutions, and the components and features of our decision support framework.^19^

To operationalise these actions, drawn from O'Cathain et al.,^19^ we extracted key ideas, themes and concepts from different data sources using a matrix approach following other similar studies^20^, ^58^ (see Table 1). This helped map data across the different sources and identify the main topics to focus upon in the decision support framework. Using this information, we adapted our published rules of thumb for acute hospitals to maximise comfort for people with dementia.^15^, ^16^ We produced prototypes using different designs to lay out key information to understand what design worked best. A summary of the synthesised information and prototypes was then presented to stakeholders in workshops and individual meetings (see Table 1 for detailed synthesis and matrix).

### 2.3 Stakeholder workshops and meetings

#### 2.3.1 Aims of the workshops

We aimed to refine and codesign a pragmatic and feasible decision support framework for hospital professionals, working with individual patients living with dementia to promote comfort.

#### 2.3.2 Stakeholders and lay members

The wider study advisory group consisted of six current and former family carers of people living with dementia comprising one stakeholder workshop. A group of four experts in social care, dementia, older adults, psychiatry and general practice made up the second workshop. A third group consisted of 10 geriatricians and an older adults' nursing team. A fourth group consisted of a frailty team (nurse and occupational therapist), and two older adult nurses. Finally, we conducted two additional workshops with people living with dementia and family carers. Key professional stakeholders including health care assistants, occupational therapists, general nurses, mental health nurses and doctors of varying levels of experience and specialities were recruited for individual professional stakeholder meetings. In total 42 stakeholders took part in codesign, this included six people living with dementia, 18 professionals, and 18 family carers.

### 2.4 Data collection

We conducted six codesign group sessions, and a series of individual sessions (2020–2023). Stakeholders also provided written feedback via email, reflecting our flexible approach to maximise participation in this process.^59^

We used a modified nominal group process^60^ in the form of structured meetings aimed at problem solving to initiate the codesign process. Generally, workshops began with a welcome and introduction along with an overview of the project and aims of the workshop/meeting. Following this, findings from synthesised data discussed above were presented to participants using PowerPoint, and stakeholders were encouraged to provide their reflections and impressions on the data, including what they felt were the priorities we should focus upon. We presented stakeholders with a prototype decision support framework. Each section of the decision support framework was presented separately with questions used to facilitate feedback and discussion about each section of the decision support framework. We took detailed notes to highlight the points made and flag these for enaction or change in the decision support framework. Following each workshop/meeting, the prototypes were refined before the next workshop or meeting with further stakeholders. Finally, in the later workshops we discussed implementation and the use of the framework in practice.

## 3 RESULTS

### 3.1 Format and flow of the decision support framework

**Figure 1:**
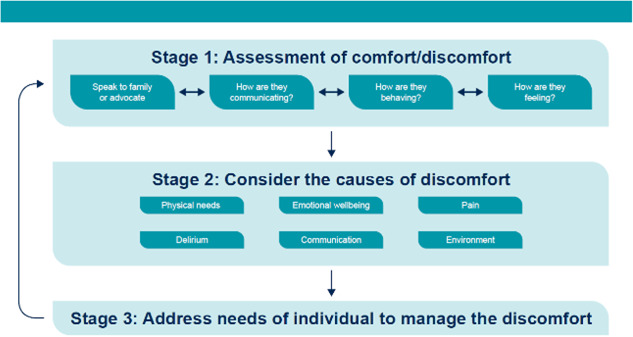
Overview of decision support framework stages.

The decision support framework operates using a three‐stage process of: (1) assessment of comfort/discomfort; (2) consider causes of discomfort; and (3) address needs of the individual to manage the discomfort (see Figure 1). It is available as a printed booklet or online flip book presented in the form of a series of flowcharts that can be used by staff easily on busy wards. Through these three stages the decision‐support framework provides a funnel structure and approach.

**Figure 2:**
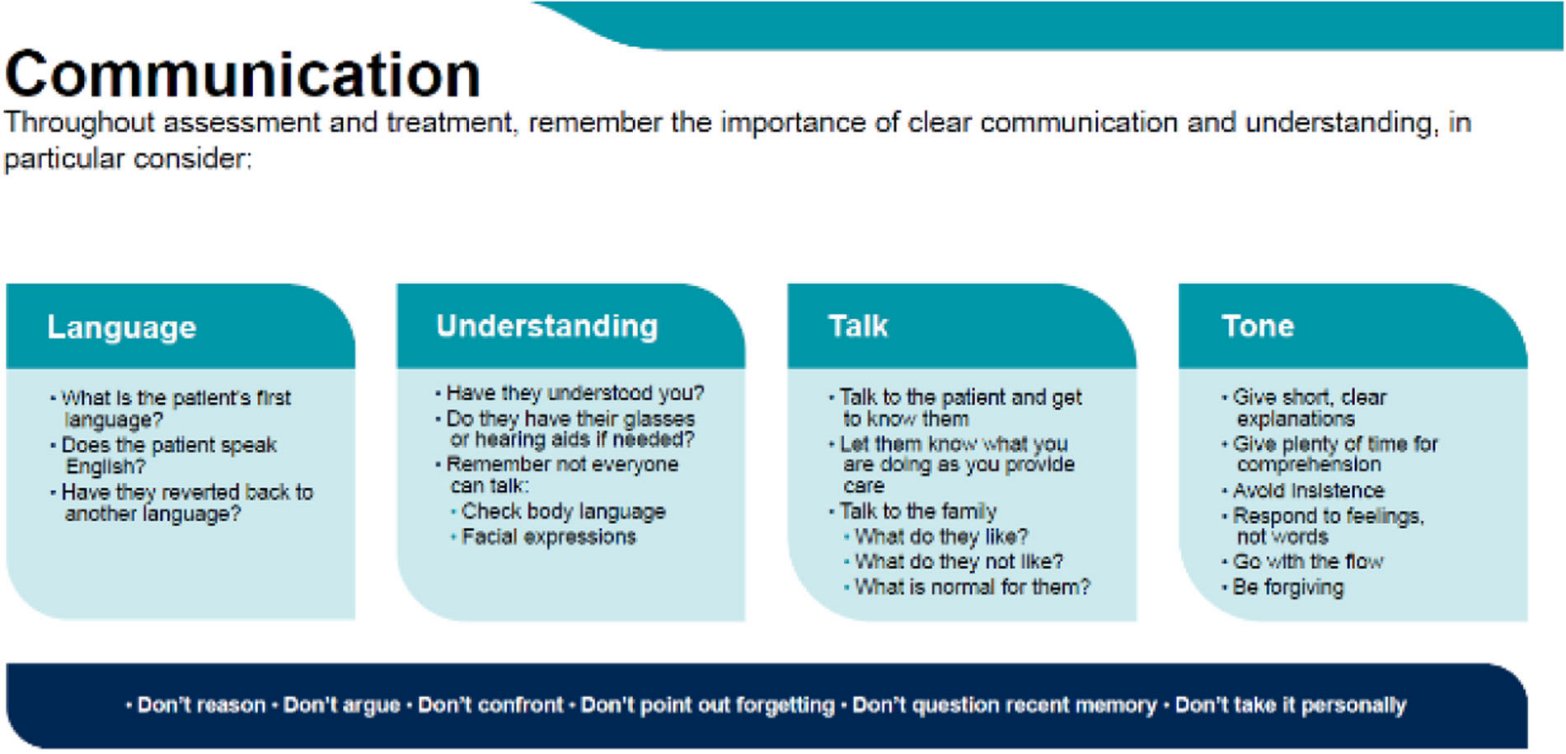
Consider communication.

The codesign groups with professionals highlighted the importance of communication not only in the sense of possible second languages, but also communication difficulties when hearing aids or dentures were misplaced or nonfunctioning. At the very start of the framework, providing an overarching message, staff are encouraged to consider the communication needs of the person living with dementia before exploring if they may be exhibiting signs of discomfort (see Figure 2). This aligns with guidance from the Mental Capacity Act (2005) and the National Institute for Health and Care Excellence Dementia guidance.^31^, ^32^ Family carers stressed the need to integrate communicating with patients with communications with families and carers to establish the patient's usual baseline. Nurses highlighted the need to understand the person's usual routine (e.g., their usual appearance or foods eaten at breakfast).

1. Go with the flow—Rather than trying to over control a situation, take a more flexible and relaxed approach for example, adapt to how the person is responding or not to you, giving them more time.
2. Be ‘forgiving’—Don't take the actions of the person with dementia personally, see the person rather than the underlying disease that drives the distress and sometimes challenging behaviour. This may be a response to the environment of the illness rather than you as a member of staff.

### 3.2 Stage 1: Assessment of comfort/discomfort

**Figure 3:**
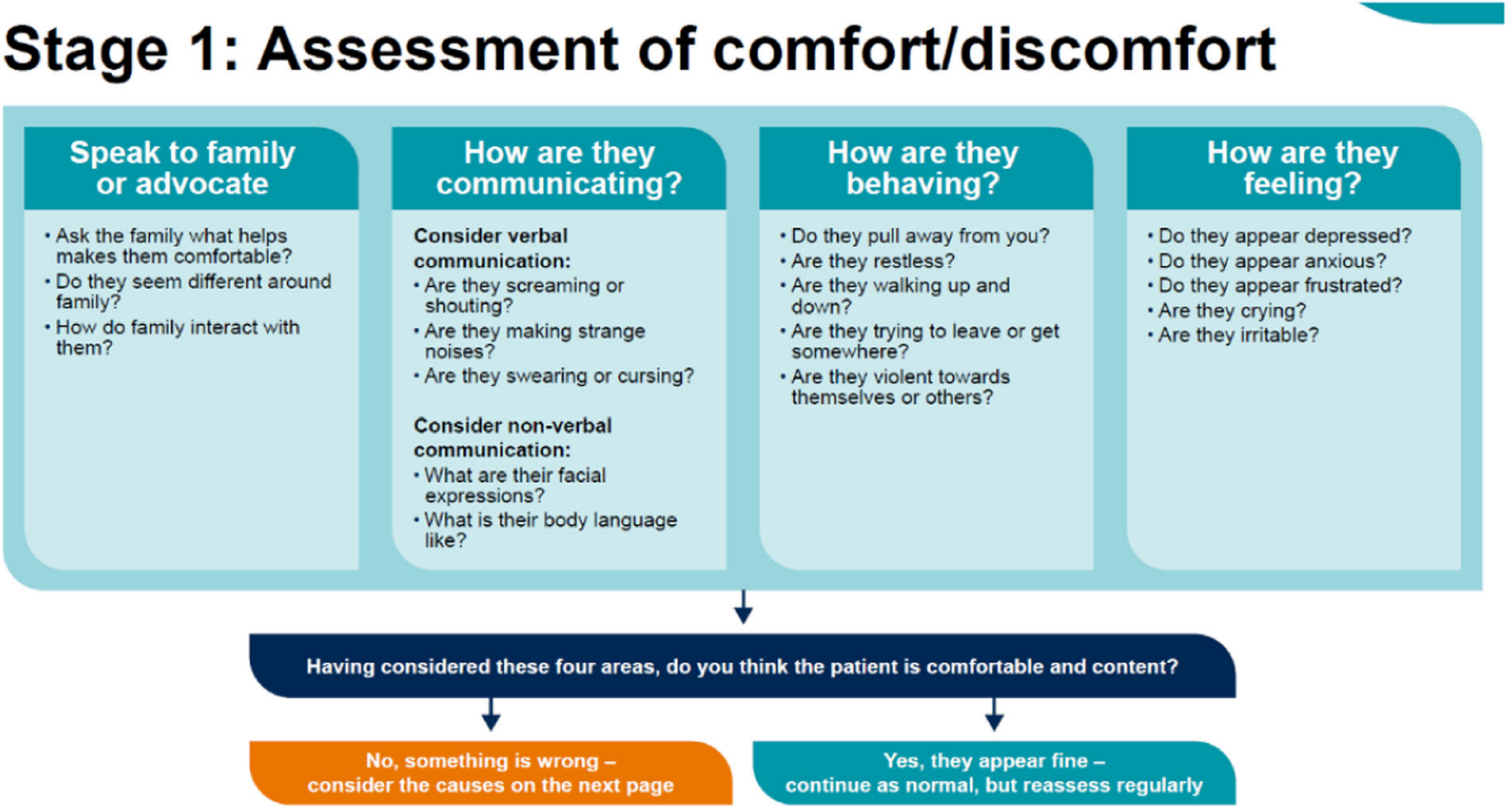
Stage 1: Assessment of comfort/discomfort.

The decision support framework prompts staff to speak to the patient's family or advocate, before considering possible causes of discomfort, to understand what is important to the patient. The Stage 1 flowchart (see Figure 3) encourages the user to assess discomfort by considering the patient's behaviour, communication and emotional state. These considerations are then distilled into a dichotomy that either classifies the patient as probably currently comfortable or in need of further help.

### 3.3 Stage 2: Consider the causes of discomfort and Stage 3: Address the need of the individual to manage the discomfort

**Figure 4:**
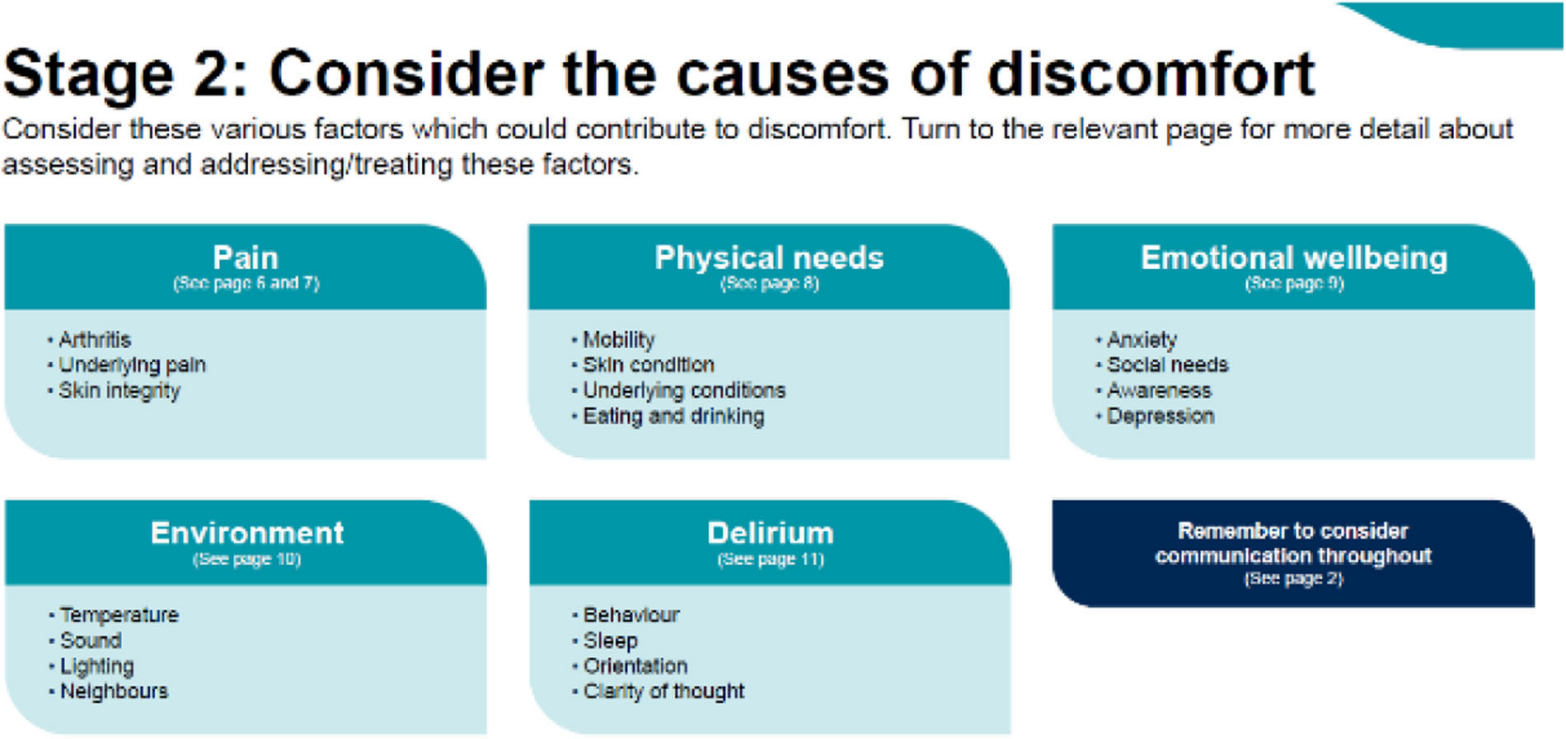
Stage 2: Overview of considerations.

**Figure 5:**
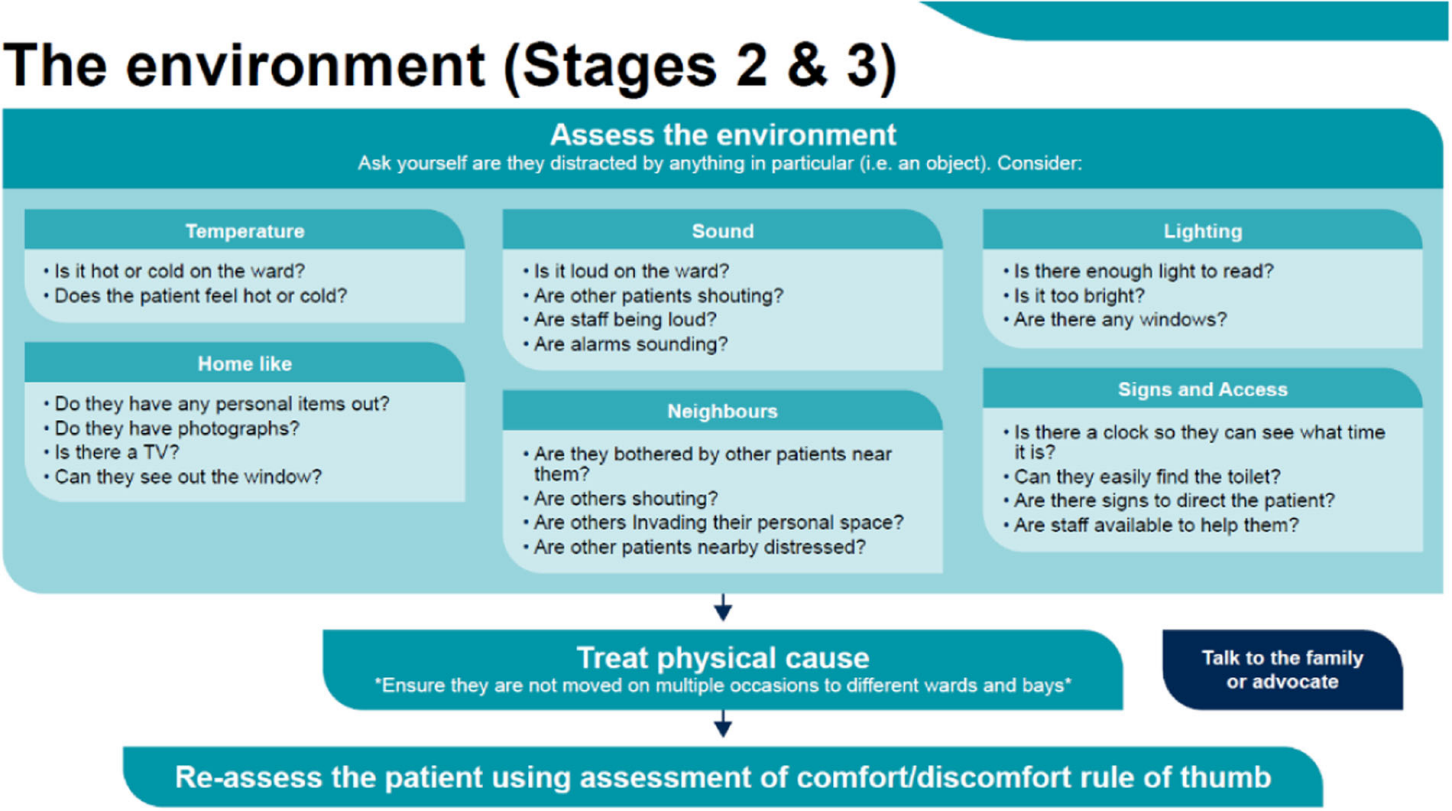
Stage 2 and 3: Consider the environment.

Following assessment, the decision support framework guides the user to consider possible causes of discomfort, using the six categories which were identified as the main areas of discomfort and comfort: physical needs, emotional wellbeing, delirium, pain, communication and the environment. We provide some overarching prompts initially (see Figure 4) and direct the user to specific additional pages that address these categories in further detail (see Figure 5; for an example of an additional page with further details).

**Figure 6:**
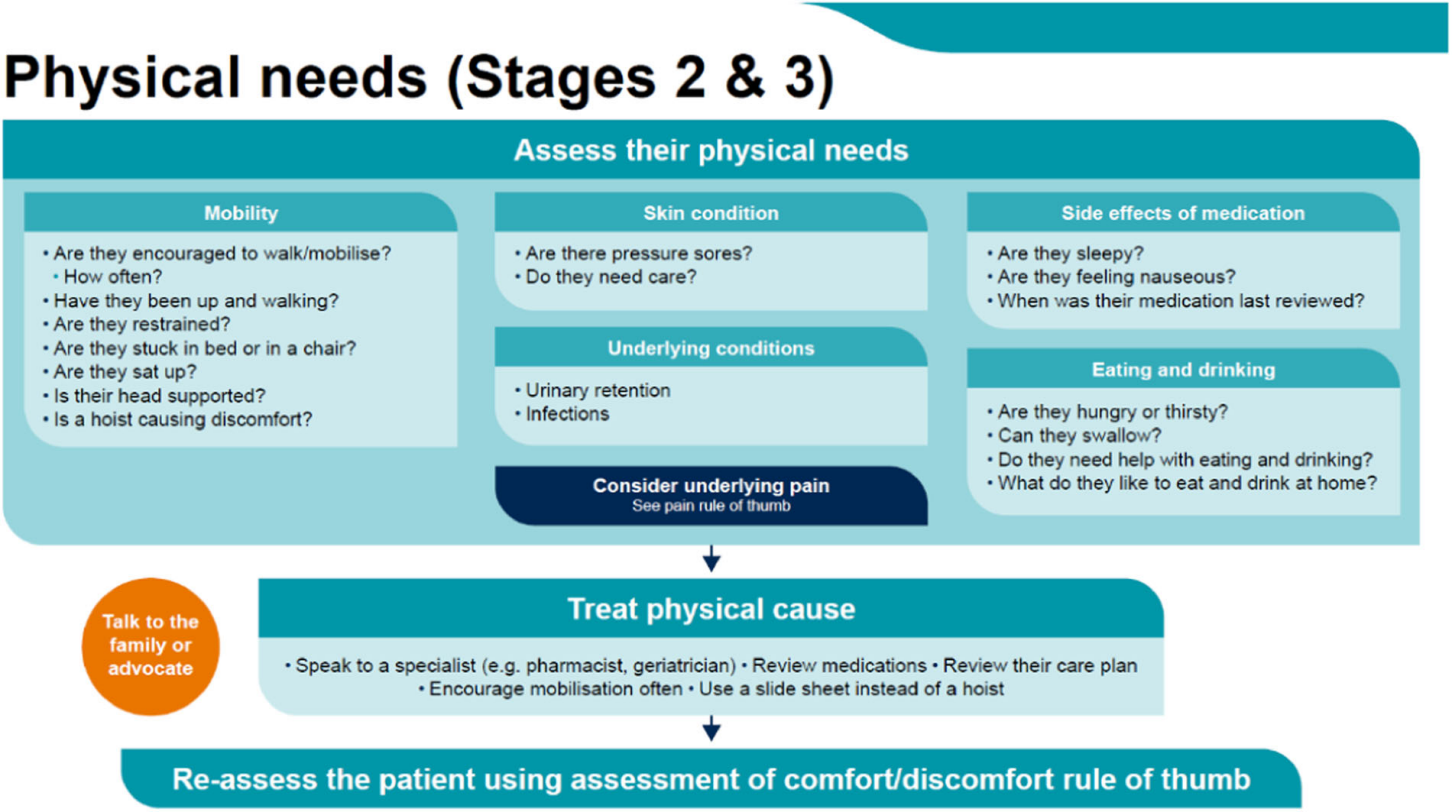
Stages 2 and 3: Consider physical needs.

From the systematic review, interviews, and the codesign process, we identified several ways to address sources of discomfort and these are provided as prompts for staff to consider. Importantly, professionals were keen to point out that not all of these may be able to be addressed. For example, the environment often cannot be changed, so it is important for the user to consider ‘what can I do?’. As shown in Figure 4 with our codesign groups, we focussed on what could realistically be done, with prompts to consider temperature, sound, lighting and neighbours. Each category page then provides further details on how to recognise and ameliorate these specific categories of discomfort—through pharmacological or nonpharmacological interventions, escalation to specialists within the hospital, addressing social, occupational and nutritional needs, and so on (see Figures 5 and 6). Each individual category page also encourages the reassessment of patients using the initial discomfort flowchart.

### 3.4 Implementation

There was a strong feeling that the guides should not just be for professionals but also for people living with dementia and families too. People living with dementia felt they should be included in decision making, and this toolkit could be used in conversations between patients and professionals. Professionals felt there was a need to not have this material on the patient's electronic record where it may be hard to find and hence use, but instead printed off and placed at the end of the bed or bay. There was a strong sense the rules of thumb needed to be visible to be used. Providing hard copies could then allow for families to view them when visiting and given the opportunity to add to them with personal details and information to help professionals when assessing comfort/discomfort or providing care. It was agreed that the rules of thumb break down complexity well, but are still fairly lengthy and so not everyone will use them. However, Figure 4 was a good example of an overview which struck a balance of enough but not too much detail, and could be used as posters on wards or at nursing stations to nudge and prompt staff to consider comfort and discomfort.

## 4 DISCUSSION

This paper presents and describes the development of a decision support framework consisting of rules of thumb, through the synthesis of diverse data and a subsequent process of codesign. This decision support framework is the first specifically directed at addressing the needs of older hospital inpatients living with dementia throughout the dementia trajectory. We have reported on a range of data collection methods that helped ensure adaptability and specificity to this patient group, as well as their family carers and the multidisciplinary ward team working with them. We believe the framework will also be beneficial to others with young onset dementia who have had a medical or surgical admission to an acute hospital, however, would need tailoring to specific needs this population may have. We have provided an overview of the development of this decision support framework, including evidence found through literature/guidelines, a quantitative study in the acute hospital environment and qualitative experiences of family carers and staff. Codesign with people with lived experience of living with or caring for those who live with dementia is a major strength of this approach and enables us to develop a framework spanning a range of situations encountered in acute hospitals that may cause distress or discomfort.

### 4.1 Distress and discomfort in dementia

Signs of distress and agitation are common in hospital inpatients living with dementia.^36^ Patients may be confused and disoriented, making it hard for them to communicate their needs. This decision support framework was aimed at elucidating and addressing factors that may be causing discomfort in people who may not be able to process or communicate this clearly. As such, the decision support framework needed to consider discomfort in a holistic sense, considering physical, emotional and social sources of discomfort and distress. This approach was influenced by Dame Cicely Saunders' notion of total pain,^61^ in which pain at the end of life is considered to contain facets of physical, emotional, social and spiritual suffering. The decision support framework prompts the observer to consider all potential needs of the patient, beyond purely ‘physical’ approaches.

### 4.2 Managing care for complex needs

Both staff and patients find addressing discomfort and distress in the acute hospital setting difficult. It is upsetting for patients, and difficult for staff to interpret. We know that pain is difficult to identify and treat because of neuropathological changes in dementia,^62^ and is associated with distress or behavioural and psychological symptoms of dementia.^63^ Professionals working in inpatient settings with patients living with dementia often report that it is difficult to manage such distress, and that professional roles may be unclear.^64^ This decision support framework aims to systematise and make clear means of amelioration and escalation for addressing sources of discomfort and distress—either before such signs manifest as severe problems, or by dealing with early signs in a way that prioritises the identification of underlying causes. However, it is also important to consider the wider ward culture where routines and processes of care are often prioritised to fill regulatory obligations.^39^ This can mean that some elements of care, such as ‘getting to know’ the individual, are missed or receive less attention. The decision support framework could be used in conjunction with considering the ward and wider hospital culture to think about what we can do for patients and how.

### 4.3 Strengths and limitations

This paper provides a comprehensive overview of the development of a decision support framework supported by diverse data sources and refined through codesign. This decision support framework was rigorously developed through codesign with end users, embedded in clinical practice and evidence from a range of sources. Combining these forms of evidence proved to be feasible and efficient as a means of building data for the codesign foundation. This diversity of data and information was a strength in both developing the framework, and of the finished framework itself. The initial cohort study and codesign elements of this development process took place under COVID‐19 social restrictions, which meant pivoting to digital and remote codesign facilitation. In practice, this worked well and had a positive effect on recruitment as no stakeholders were unable to participate due to travel times or access needs, as found in similar work during COVID‐19.^65^ However, this strategy may have excluded others lacking digital literacy or access to computers or smartphones. We also acknowledge that our staff interviews were limited in number and in the breadth of personnel interviewed, for example, we did not interview general nurses or health care assistants who are the staff in most regular contact with patients. However, in our stakeholder workshops we ensured we included general nurses and a range of different stakeholders.

### 4.4 Implications for research and practice

We have developed a decision support framework which is ready for testing and implementation. Research findings, especially in dementia interventions, often face delays in reaching practice, creating a translational gap.^66^ There has been a lack of focus on the translation of dementia research into practice, with an urgent call to address this.^67^ Our stakeholders have identified implementation as a key next step. The framework is based on best evidence and good practice, codesigned with experts therefore the focus should be on evaluating the implementation rather than traditional measures of effectiveness. We propose an implementation or hybrid study exploring implementation/effectiveness in acute hospital settings with hospital staff and family carers is the next step. Key to implementation is understanding how such toolkits are used. As the codesign groups highlight, visibility is key to engaging users, uploading the toolkit to a digital system or digital health records may make the toolkit less visible. A further key consideration to future use is time, with some stakeholders highlighting the framework may be too long for use by some. The nature of the rules of thumb is that they are brief and prompt thinking rather than producing further lengthy guidance which is currently available in hospitals (national and local guidance), rather they are a concise synthesis of best practice.^15^ Alternative solutions include the use of large posters on hospital wards to prompt staff, a toolkit printed with opportunities for patients/families to access, tailor and personalise it to support staff when providing care. The rules of thumb have been designed to be simple to use requiring no formal training and minimal explanation, but simply provide support through decision making processes. However, it is also possible that the rules of thumb have the potential to be used as a component of a larger programme of education and training about dementia care in acute hospitals, but not a requirement for use. To ensure families are aware of the rules of thumb and how they can be used, it is important that staff in hospitals raise awareness of the rules of thumb for families and patients, providing them with the opportunity to read through and think about any personal additions they could make to them which would benefit staff decision making.

## 5 CONCLUSIONS

This process of framework‐building led to an evidence‐based support document that reflects the needs and experiences of people living with dementia, family carers and professionals. Thus, synthesising diverse quantitative and qualitative evidence to build support frameworks is a feasible approach to better address the needs of patients living with dementia in the acute hospital setting. The result is a framework which is now ready for evaluation and implementation.

## AUTHOR CONTRIBUTIONS

Nathan Davies, Greta Rait, Victoria Vickerstaff, Jill Manthorpe, Jane Ward and Elizabeth L. Sampson conceived the idea and design of the study. Nathan Davies, Greta Rait, Victoria Vickerstaff, Jill Manthorpe, Jane Wilcock, Jane Ward and Elizabeth L. Sampson acquired funding. Emily West, Ellen McCloy Smith, Nathan Davies, Elizabeth L. Sampson, Victoria Vickerstaff and Malvi Shah acquired data, and contributed to the analysis. All authors contributed to the interpretation of the results. All authors drafted and finalised the manuscript. All authors have approved the version to be published and agree to be accountable for all aspects of the work.

## CONFLICT OF INTEREST STATEMENT

The authors declare no conflict of interest.

## ETHICS STATEMENT

Health Research Authority approval was provided by London Queens Square on 25 March 2019 (19/LO/0036). All research participants provided written or verbal informed consent.

## Data Availability

The data that support the findings of this study are available from the corresponding author upon reasonable request.
